# Comparative genomic epidemiology of serotype 3 IPD and carriage isolates from Southampton, UK between 2005 and 2017

**DOI:** 10.1099/mgen.0.000945

**Published:** 2023-03-03

**Authors:** David W. Cleary, Stephanie W. Lo, Narender Kumar, Stephen D. Bentley, Saul N. Faust, Stuart C. Clarke

**Affiliations:** ^1^​ Institute of Microbiology and Infection, College of Medical and Dental Sciences, University of Birmingham, Edgbaston, Birmingham, UK; ^2^​ Faculty of Medicine and Institute for Life Sciences, University of Southampton, Southampton, UK; ^3^​ Parasites and Microbes, Wellcome Sanger Institute, Hinxton, UK; ^4^​ NIHR Southampton Biomedical Research Centre, University Hospital Southampton Foundation NHS Trust, Southampton, UK; ^5^​ Southampton Clinical Research Facility, University Hospital Southampton Foundation NHS Trust, Southampton, UK; ^6^​ Global Health Research Institute, University of Southampton, Southampton, UK

**Keywords:** serotype 3, IPD, invasive pneumococcal disease, *Streptococcus pneumoniae*, carriage

## Abstract

Serotype 3 pneumococci remains a significant cause of disease despite its inclusion in PCV13. Whilst clonal complex 180 (CC180) represents the major clone, recent studies have refined the population structure into three clades: Iα, Iβ and II, with the last being a recent divergent and more antibiotic-resistant. We present a genomic analysis of serotype 3 isolates from paediatric carriage and all-age invasive disease, collected between 2005 and 2017 in Southampton, UK. Forty-one isolates were available for analysis. Eighteen were isolated during the annual cross-sectional surveillance of paediatric pneumococcal carriage. The remaining 23 were isolated from blood/cerebrospinal fluid specimens at the University Hospital Southampton NHS Foundation Trust laboratory. All carriage isolates were CC180 GPSC12. Greater diversity was seen with invasive pneumococcal disease (IPD) with three GPSC83 (ST1377: *n*=2, ST260: *n*=1) and one GPSC3 (ST1716). For both carriage and IPD, Clade Iα was dominant (94.4 and 73.9 % respectively). Two isolates were Clade II with one from carriage (a 34-month-old, October 2017) and one invasive isolate (49-year-old, August 2015). Four IPD isolates were outside the CC180 clade. All isolates were genotypically susceptible to penicillin, erythromycin, tetracycline, co-trimoxazole and chloramphenicol. Two isolates (one each from carriage and IPD; both CC180 GPSC12) were phenotypically resistant to erythromycin and tetracycline; the IPD isolate was also resistant to oxacillin.In the Southampton area, carriage and invasive disease associated with serotype 3 is predominantly caused by Clade Iα CC180 GPSC12.

## Introduction

The post-pneumococcal conjugate vaccine (PCV) era has been characterized by a remarkable global reduction in *Streptococcus pneumoniae *-associated morbidity and mortality [1–5]. There remains, however, a significant burden of both invasive (bacteraemia, meningitis, septicaemia) and non-invasive disease (pneumonia, otitis media). Although in part driven by serotype replacement [6–9] and the rise of non-vaccine serotype (non-VT) disease [10], a persistent challenge are those vaccine-type serotypes (VT) which have proven recalcitrant to immunization programmes. Serotype 3 is one such example.

Despite its inclusion in the 13-valent PCV (PCV13), serotype 3 remains a particularly significant cause of disease globally [11]. Following PCV13 introduction in the UK in 2010 serotype 3 has continued to circulate in paediatric carriage [7, 12]. Vaccine effectiveness has been questionable given the fluctuating, albeit relatively low level, incidence of invasive pneumococcal disease (IPD) in children <5 years old [10]. Importantly, serotype 3 remains a significant burden in adult disease causing, for example, 57 % of pneumococcal community-acquired pneumonias between 2013 and 2018 [13] and 65 % of IPD in those aged >65 years old [10]. In the UK this group is offered the Pneumococcal Polysaccharide Vaccine (PPV). Lower vaccine efficacy against serotype 3 has been shown to derive from extensive capsule release, a by-product of the way in which the capsular polysaccharide is not covalently anchored to the cell surface, which prevents antibody-mediated opsonophagocytosis [14].

Whilst clonal complex 180 (CC180) represents the major clone, recent studies have refined the population structure of serotype 3 pneumococci into three clades: Iα, Iβ and II, with the last being a recent divergent and characterized as more antibiotic-resistant [15, 16]. However, there is no evidence to link this changing epidemiology to PCV13 introduction [15]. In the present study, we aimed to build upon these data and present the genomic analysis of serotype 3 isolates from a serial, cross-sectional paediatric carriage study between 2005 and 2017 in Southampton, UK, in addition to temporally and geographically concomitant isolates from all-age invasive disease.

## Methods

### Isolate collection

Carriage isolates were obtained using nasopharyngeal swabs collected from children aged 4 years or under each year commencing in the winter (October to March) of 2006/07 and for each consecutive year until 2017/18. Parents/guardians were approached for informed consent either prior to or following their child’s appointment in an outpatient department of Southampton General Hospital. Aside from age, the only other exclusion criterium was that only one child per family was swabbed. Nasopharyngeal Rayon tipped Transwabs (Medical Wire) in charcoal Amies media were used for swabbing and then plated onto Columbia Colistin Naladixic Acid agar (CNA; Oxoid) within 9 h of swabbing. IPD isolates from blood or cerebrospinal fluid (CSF) specimens were isolated in the Public Health England (PHE) laboratory at University Hospital Southampton NHS Foundation Trust between July 2005 and June 2017.

### Pneumococcal growth and confirmation

Confirmation of presumptive *S. pneumoniae* was done on Columbia Blood Agar (CBA; Oxoid) using optochin sensitivity indicated by a ≥14 mm diameter inhibition zone around the disc (Thermo Scientific). Only one colony of *S. pneumoniae* per participant was selected for further analysis.

### Whole genome sequencing

Isolates from STGG (skim milk, tryptone, glucose, and glycerin) stocks were cultured on CNA plates and incubated overnight at 37 °C in 5 % CO_2_. Genomic DNA extraction was carried out using a QIAamp DNA mini kit (Qiagen) according to the manufacturer’s instructions. The DNA extracts were sent to the Wellcome Sanger Institute (WSI) for whole genome sequencing (WGS) using Illumina HiSeq or 10X platforms generating initially 2×75 bp, 2×100 bp and later 2×150 bp paired-end reads from libraries prepared using TruSeq chemistry.

### Bioinformatic analysis

The processing of WGS data has been previously described [17] where serotype is derived using SeroBA [18] and sequence type using multilocus sequence typing (MLST) [19]. Global Pneumococcal Sequence Clustering (GPSC) for each isolate was done using Kmer-based clustering in popPUNK [20]. Likewise antibiotic resistance profiles were generated using previously described methods [21–23], including penicillin (encoded by the genes *pbp1A*, *pbp2B* and *pbp2A*), chloramphenicol (*cat*), cotrimoxazole (*folA* and *folP*), erythromycin (*ermB* and *mefA*), fluoroquinolones (*gyrA* and *parC*), tetracycline [*tet*(M), *tet*(O) and *tet*(S/M)] and vancomycin (*vanA* and *vanB*). Phylogenies were made using nextflow v21.04.1 nf-core/bactmap v1.0.0 [24]. Briefly, reads were mapped to a serotype 3 ST180 strain OXC141 (accession: NC_017592.1) reference with bwa mem [25], and alignments were indexed and sorted using samtools v1.10 [26] with variants called and filtered using bcftools v1.11 [27]. Recombinogenic regions were then identified and removed using Gubbins v2.4.1 [28] and non-variable sites removed using snp-sites v2.5.1 [29]. Finally, a maximum-likelihood tree was built using RAxML-NG v1.0.2 with the GTR+Gamma model of nucleotide substitution [30]. Recombination regions were visualized using Phandango [31]. Clade designation was determined by placement of isolates within the phylogeny previously generated by Azarian *et al*. [15] using the method described above. Temporal analysis of the CC180 isolates was done using BactDating v1.1.0 [32] using the *aligned_pseudogenomes* output from Gubbins with the number of Markov chain Monte Carlo (MCMC) iterations set to 10 000 (nbIts=1e4). Protein antigen detection as previously described [15] was done using both ABRicate v1.0.1 [33] and SRST2 v0.2.0 [34] with a reference sequence database kindly donated by Dr Taj Azarian. The database contains 61 alleles of 13 antigens (*pspC n*=18, *pspA n*=12, *nanA n*=3, *phtD n*=2, *ply n*=2, *zmpA n*=7, *rrgA n*=3, *rrgB n*=3, *rrgC n*=3, *stkP n*=2, *strH n*=2, SP0609 *n*=2 and SP2194 *n*=2). Default parameters were used throughout, apart from with SRST2 and both *pspC* and *pspA* where the coverage threshold was reduced to 80 % and maximum divergence increased from 10% (default) to 20 % to account for the greater diversity in UK collections as described by Groves *et al*. [16].

### Compute resources

Nextflow nf-core/bactmap was implemented on the Cloud Infrastructure for Microbial Bioinformatics (CLIMB) resource [35]. The Iridis HPC at the University of Southampton was used for all other compute requirements.

### Statistics and data visualization

All statistical analysis and data visualization was done in R v4.2.1 [36] using RStudio v2021.09.2 [37] with graphics built using the grammar of the graphics package ggplot v3.3.5 [37] and the phylogenetic tree extension ggtree v3.2.1 [38].

### Data availability

All sequencing data (fastqs) have been deposited in the European Nucleotide Archive under study accession PRJEB2417 (‘Whole genome sequencing of carried *Streptococcus pneumoniae* during the implementation of pneumococcal conjugate vaccines in the UK’) and PRJEB6332 (‘Identifying pneumococcal genetic determinants for progression from colonisation to serious disease’).

## Results and discussion

Following the global emergence of Clade II CC180 serotype 3 [15] there is a pressing need to examine the epidemiology of this serotype, particularly given that it continues to be a burden of invasive disease. Notwithstanding the important focus on disease, these analyses must also include the use of isolates derived from episodes of carriage [39]. To this end we gathered isolates collected from the Southampton area in the UK, collected over a 12 year period, to undertake a genomic analysis of population structure, antimicrobial resistance and the distribution of important virulence-associated antigens.

**Fig. 1.**
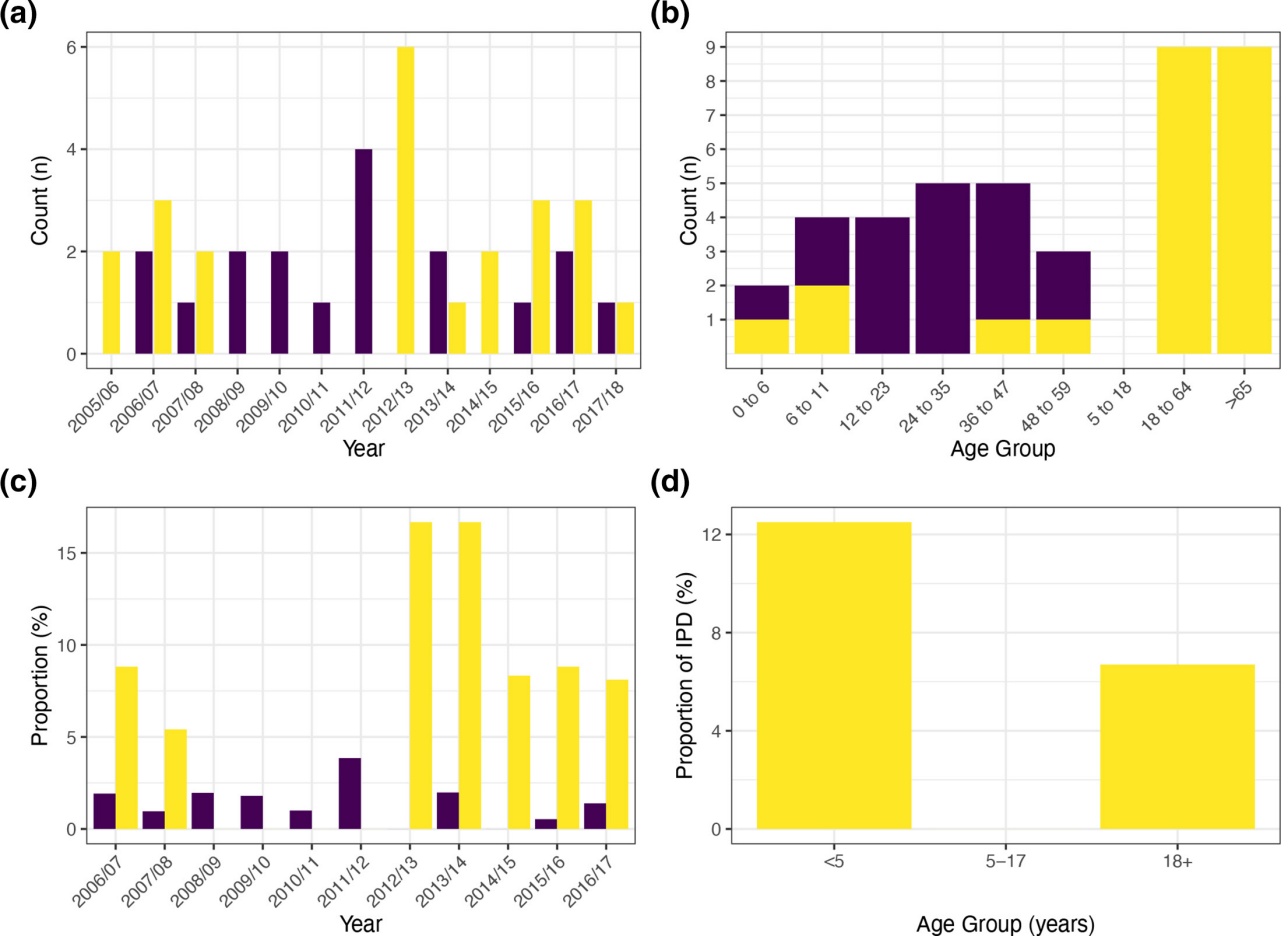
Isolation period of *S. pneumoniae s*erotype 3 isolates (**a**) with carriage isolates shown in purple and IPD in yellow. The split between age groups is shown in (b) where all carriage isolates (*n*=18) were from children <5 years old, compared to just 21.7 % (*n*=5/23) of IPD isolates. Serotype 3 as a proportion of all *S. pneumoniae* sampled each period is shown in (c), with the proportion that accounted for IPD by age group in (d).

Between 2005 and 2017, *n*=18 isolates of serotype 3 pneumococci were isolated from healthy children under the age of 5 years. No carriage isolates were identified in 2012/13 or 2014/15 (the absence in 2005/06 is a consequence of this pre-dating the start of the paediatric carriage study which began in October 2006 i.e., the 2006/07 winter period) (Fig. 1a). A similar number of IPD isolates (from children and adults) were available (*n*=23) (Table 1; Fig. 1a). No serotype 3 pneumococci were isolated from IPD cases in 2008/09, 2009/10, 2010/11 or 2011/12 (Fig. 1). The highest number of isolates from IPD was seen in 2012/13 when six cases of serotype 3-associated disease were recorded. All age groups of <5-year-olds had at least one episode of carriage. In contrast, of the 21.7 % (*n*=5/23) of IPD cases that occurred in children under 5 years old, three cases were between the ages of 0 and 11 months (average age: 3 months) with the remaining two being from a 3- and a 4-year-old (Table 1; Fig. 1b). On average the proportion of serotype 3 observed in carriage has remained relatively steady at 1.4 %, ranging from 0 to 3.9 %, which was observed in the 2011/12 winter period (Fig. 1c). This contrasts with other PCV7 and PCV13 serotypes that have decreased over the same period [40]. For IPD the proportion of serotype 3 ranged from 0 % (all periods between 2008/09 and 2011/12) to a maximum of 16.7 % (average: 6.6 %) (Fig. 1c). Five of the six highest serotype 3-related IPD periods have occurred since the introduction of PCV13. The majority of IPD was in the >65-year-old group (39.1 %; *n*=9/23; average age: 83 years) followed by the 50–64-year-olds (26.1 %; *n*=6/23; average age: 59 years). However, proportionally serotype 3 accounted for 12.5 % of IPD in children <5 years (*n*=5/40) compared to 6.7  % of adults >18 years (*n*=18/274) (Fig. 1d).

**Table 1.** Age distribution and genotypes of serotype 3 *Streptococcus pneumoniae* from paediatric carriage (*n*=18) and all-age invasive pneumococcal disease (IPD, *n*=23) from Southampton, UK, between 2005 and 2017

	Carriage (*n*=18)	IPD (*n*=23)
Age (years)	*N* (%)	*N* (%)
0–5	1 (5.6)	1 (4.3)
6–11	2 (11.1)	2 (8.7)
12–23	4 (22.2)	− (−)
24–35	5 (27.8)	− (−)
36–47	4 (22.2)	1 (4.3)
48–59	2 (11.1)	1 (4.3
5–17	− (−)	− (−)
18–49	− (−)	3 (13.0)
50–64	− (−)	6 (26.1)
65+	− (−)	9 (39.1)
MLST*	ST180: 18 (100)	ST180: 19 (82.6); ST1377: 2 (8.7); ST260: 1 (4.3); ST17176: 1 (4.3)
GPSC†	GPSC12: 18 (100)	GPSC12 : 19 (82.6); GPSC83: 3 (13.0); GPSC3: 1 (4.3)
CC180 Clade	Iα: 17 (94.4); II: 1 (5.6)	Iα: 18 (73.9); II: 1 (4.3); none: 4 (17.4)

*MLST: multi-locus sequencing type.

†GPSC: Global Pneumococcal Sequence Cluster.

The increase in proportion of serotype 3-associated IPD between 2012/13 and 2016/17 matches generally that observed across the UK [16], although 2012/13 was notably higher. It is tempting to consider a link between the higher carriage observed in the previous winter period (2011/12) with this high level of adult disease. Although such a relationship between paediatric carriage and adult IPD has been noted elsewhere, the model was shown not to account for the disease burden associated with serotype 3 [41], particularly in adults over 40 years which accounts for 94 % of the adult IPD presented in this study. Additional caution should also come from considering the small numbers involved and that stochastic fluctuations due to this being a local convenience collection may better explain this observation.

Genomically, all carriage isolates were CC180 (ST180), GPSC12, with 82.6 % (*n*=19/23) of IPD isolates also belonging to this same clonal complex and sequence cluster. The remaining four IPD isolates were split between GPSC83 (*n*=3; ST1377 and 260) and GPSC3 (*n*=1, ST17176). GPSC3 serotype 3 is not a common combination, representing only 1.1 % (*n*=4/358) of previously described isolates in this sequence cluster [17]; ST17176 had also not been described previously. All four of these previously described GPSC3 serotype 3 isolates were isolated from cases of disease, with two from South Africa (isolated in 2005 and 2014 respectively) and one each from Trinidad and Tobago (1997) and Qatar (2014) [17]. It is worth noting, however, that GPSC3 does include invasive serotypes 8 and 33F and has been flagged as a lineage that causes significant non-VT disease since the introduction of PCV13 [42]. In contrast, all GPSC83 described so far have been serotype 3 (87.0 %, *n*=20/23; the remaining three having inconclusive serotype designations), with all but two from carriage and found globally in Africa, Asia, Europe, and both North and South America [17]. ST 260 is also the most common sequence type of GPSC83 (34.8%, *n*=8/23) with ST1377 only found twice previously [17]. The former has, however, been found sporadically within other serotypes (14, 6B, 7F and 9V) but these are single incidences and represent <5 % of publicly available data [43].

**Fig. 2.**
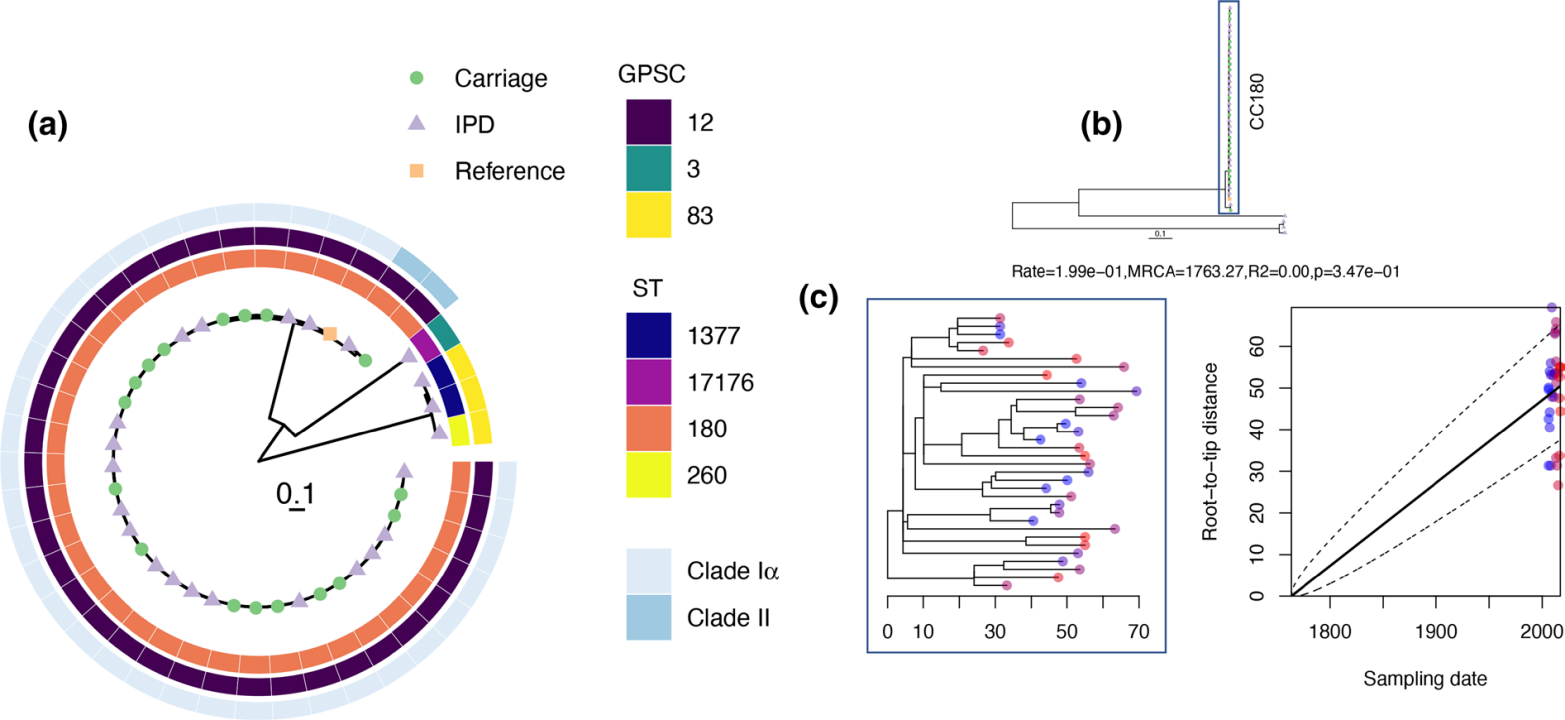
(a) Maximum-likelihood phylogeny of all serotype 3 pneumococci generated using RAxML-NG on a recombination-corrected alignment done using Gubbins. Tree tips are coloured according to various study groups: carriage (green circle), IPD (purple triangle) and the reference (OXC141; NC_017592.1). Associated metadata are shown as coloured rings depicting ST (inner), GPSC (middle) and Clade (outer). The scale bar indicates the estimated average number of substitutions per site. (b) The same phylogeny with CC180 isolates highlighted by the blue box from which a time-corrected phylogeny (**c**) was reconstructed using BactDating where blue tips show earliest isolate collections and red the most recent.

**Fig. 3.**
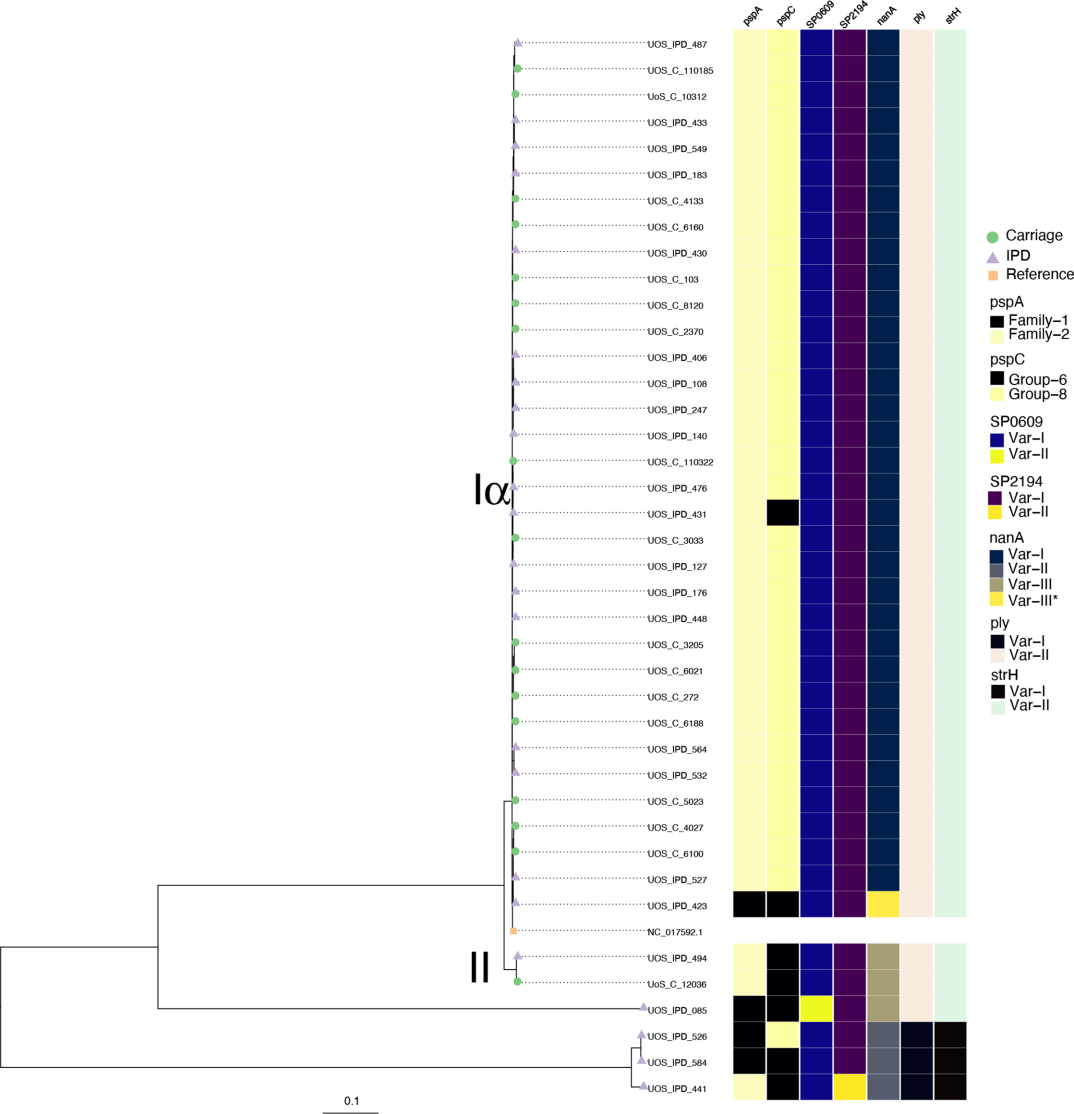
Maximum-likelihood phylogeny of serotype 3 pneumococci with distribution of variable antigens. Tree tips are coloured according to various study groups: carriage (green circle), IPD (purple triangle) and the reference (OXC141; NC_017592.1). The scale bar indicates the estimated average number of substitutions per site.

**Fig. 4.**
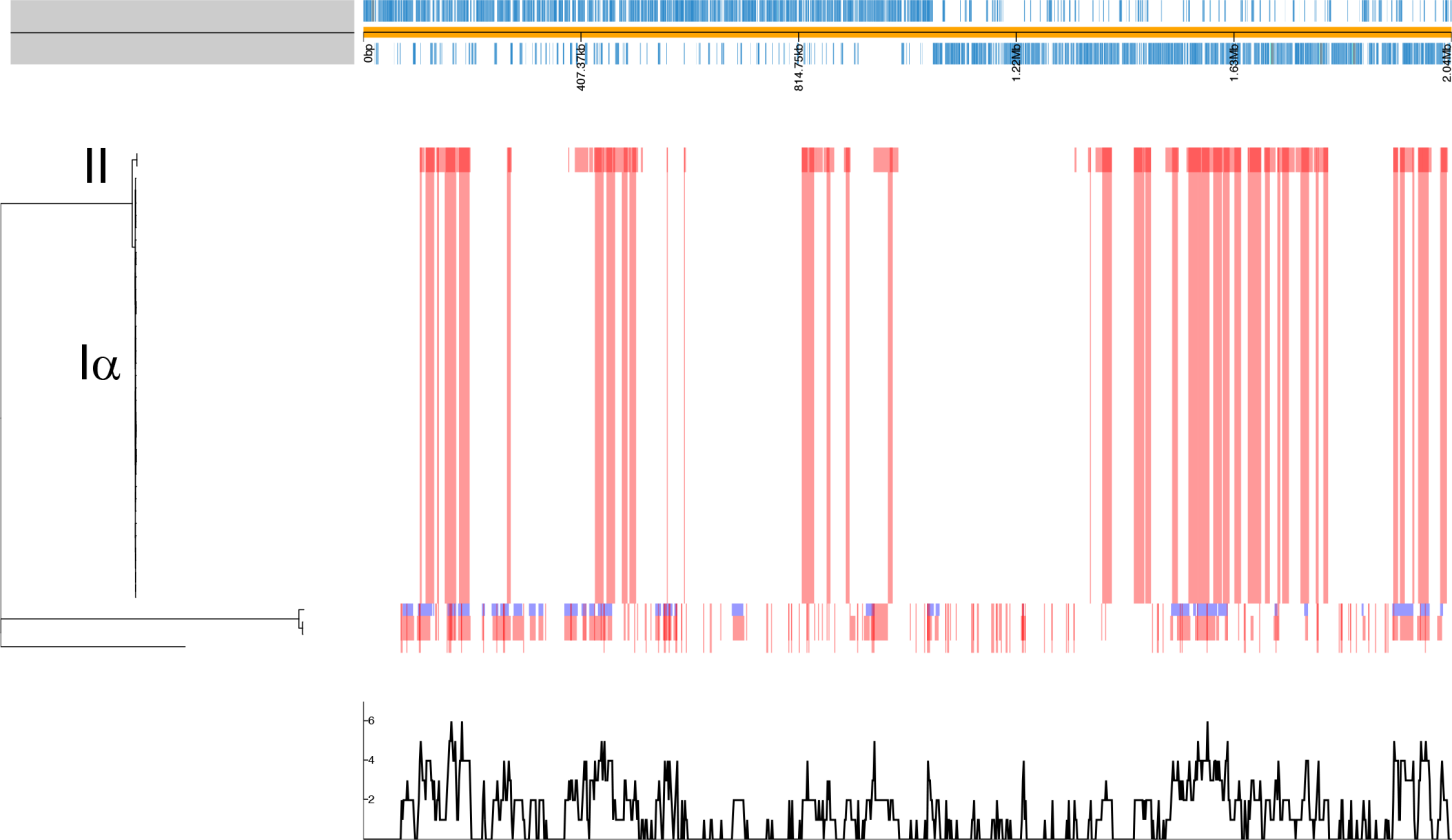
Maximum-likelihood phylogeny of all serotype 3 isolates generated using RAxML-NG with clades Iα and II shown (left) with a linear genome map of the reference *S. pneumoniae* OXC141 (top). Recombination events are shown as red and blue blocks, with red indicating a recombination event that has occurred on an ancestral branch and blue showing recombination blocks unique to that terminal node, i.e., isolate. The bottom plot shows the cumulative frequency of recombination at positions along the reference.

No phylogenetic distinction between carriage and IPD isolates was seen for CC180 GPSC12 isolates in which *n*=17/18 carriage and *n*=18/19 IPD were assigned to Clade Iα (Fig. 2a). The absence of Iβ is unsurprising and in keeping with previous data given that it is the most infrequently observed both globally [15] and in England and Wales [16]. Despite the apparent close phylogenetic relatedness of the Clade Iα isolates (Fig. 2b), there was no evidence for transmission between study participants nor for a temporal signal (Fig. 2c). Previously, Clade II had been shown to be rapidly increasing in IPD in England and Wales [16] although it appears not be a consequence of narrow vaccine efficacy [15, 44]. That said, we note that three of the four isolates that could not be assigned to a clade were recovered post-PCV13 and were all from cases of invasive disease, perhaps suggesting an increase in the diversity of serotype 3 post-PCV13. Regardless, antigenic variation and antimicrobial resistance within this diverging Clade II population has been raised as a potential cause of its emergence. In contrast, only one disease isolate of this Clade II was observed in this study, recovered from a 49-year-old in 2015. We did not see a significant shift in carriage as has been seen elsewhere [44] with only one isolate recovered from a 34-month-old in 2017. The dates of isolation (post-PCV13) are in keeping with the emergence of Clade II, but with limited numbers it is difficult to determine the extent of this replacement. What is interesting is that higher levels of serotype 3 IPD observed in Southampton is despite the absence of Clade II. Examining the diversity of certain pneumococcal antigens however does support the idea of diversity being a factor in emergence of Clade II. Some antigens consistently belonged to the same variants regardless of Clade such as *pspA* (pneumococcal surface protein A; with one exception as described below) where all Clade Iα and II isolates had Family 2 variants (Fig. 3). Other examples included SP0609 (an amino acid ABC transporter), SP2194 (ATP-dependent Clp protease), *ply* (pneumolysin) and *strH* (Beta-*N*-acetylhexosaminidase) (Fig. 3). Conversely, there were antigens absent as expected in serotype 3 isolates (i.e. *rrgABC*; pilus subunit). Nevertheless, diversity was seen for *pspC* where 32/34 Clade Iα isolates were Group 6 and both Clade II were Group 8. This is entirely in keeping with that described previously [16]. The two Clade Iα isolates which possessed a Group 8 *pspC* are perhaps more unexpected. Both were isolated in 2013 from adult invasive disease. Isolate UOS_IPD_423 was also variant for *pspA* (Family 1) and *nanA* which had, unlike the other Clade Iα isolates, a variant of Var-III – the type found in both Clade II isolates. The *pspC* and *nanA* antigen designations suggest this isolate is, antigenically at least, more akin to a Clade II isolate than Clade Iα. Looking at recombination within the clade and on the terminal nodes representing this isolate, however, did not shed any light on these differences (Fig. 4). As previously described Clade II was more recombinogenic at the internal, ancestral node compared to Clade Iα. Here the relative impacts of recombination to mutation (*r/m*) were 20.0 and 8.7, and the relative rates of recombination to mutation (*ρ/θ*) were 0.181 and 0.112 respectively. No terminal node recombination was detected for the Clade II isolates, which is not surprising given there are only two. However, terminal node recombination statistics for Clade Iα (*r/m*=0.02 and *p/θ* = 0.003) were consistent with those observed previously (*r/m*=0.07 and *ρ/θ*=0.001 [15]).

All isolates were shown to be genotypically susceptible to penicillin, erythromycin, tetracycline, co-trimoxazole and chloramphenicol. Two isolates (one each from carriage and IPD; both CC180 GPSC12) were phenotypically resistant to erythromycin and tetracycline; the IPD isolate, which was isolated in 2014, was also resistant to oxacillin.

Whilst this study provides useful data on the epidemiology and population structure of an important pneumococcal serotype, there are limitations. From a carriage perspective, the population examined represents a convenience sample from a study that was not powered to specifically detect serotype 3. The paediatric carriage study was powered to enable the detection of an estimated 50 % relative reduction in carriage following PCV7 introduction with 80 % power at a 5 % significance level. This meant a minimum of 100 pneumococcal isolates were collected each year; with the infrequency of colonization this snapshot collection is not an accurate picture of serotype 3 carriage in this community. Further, no carriage sampling was undertaken in an adult population. This would be a much-needed addition to the current study, as it might reveal a serotype 3 expansion across demographics which could explain the increase in adult IPD in the absence of notably increased carriage in children. The IPD isolates also represent a convenience sample taken from one hospital, albeit a large regional centre, and therefore may not be representative of the national picture.

### Conclusion

Serotype 3 carriage and invasive disease episodes were identified throughout the study period, in both infants and adults. This adds further support to the notion of lower PCV13 effectiveness against this serotype. The serotype 3 epidemiology around Southampton is driven by a closely related Clade Iα CC180 GPSC12 pneumococcal population. Although we did not see a large transition between Clade Iα and Clade II, the fact that we did recover the latter may reflect a shift and continued surveillance will determine if this is an expansion as seen elsewhere.
